# Economic Evaluation of Community-Based HIV Prevention Programs in Ontario: Evidence of Effectiveness in Reducing HIV Infections and Health Care Costs

**DOI:** 10.1007/s10461-015-1109-8

**Published:** 2015-07-08

**Authors:** Stephanie K. Y. Choi, David R. Holtgrave, Jean Bacon, Rick Kennedy, Joanne Lush, Frank McGee, George A. Tomlinson, Sean B. Rourke

**Affiliations:** The Ontario HIV Treatment Network, Toronto, Ontario Canada; Faculty of Medicine, University of Toronto, Toronto, Ontario Canada; Li Ka Shing Knowledge Institute of St. Michael’s Hospital, 30 Bond St., Toronto, Ontario M5B 1W8 Canada; Department of Health, Behaviour and Society, Bloomberg School of Public Health, John Hopkins University, Baltimore, MD USA; The Ontario AIDS Network, Toronto, Ontario Canada; AIDS Bureau, Ontario Ministry of Health and Long-term Care, Toronto, Ontario Canada; Institute of Health Policy, Management and Evaluation, University of Toronto, Toronto, Ontario Canada; Division of Biostatistics, Dalla Lana School of Public Health, University of Toronto, Toronto, Ontario Canada; University Health Network, Toronto, Ontario Canada; Department of Psychiatry, Faculty of Medicine, University of Toronto, Toronto, Ontario Canada

**Keywords:** Macro-level analysis, Program evaluation, HIV, Primary prevention, Community action, Return on investment

## Abstract

Investments in community-based HIV prevention programs in Ontario over the past two and a half decades are assumed to have had an impact on the HIV epidemic, but they have never been systematically evaluated. To help close this knowledge gap, we conducted a macro-level evaluation of investment in Ontario HIV prevention programs from the payer perspective. Our results showed that, from 1987 to 2011, province-wide community-based programs helped to avert a total of 16,672 HIV infections, saving Ontario’s health care system approximately $6.5 billion Canadian dollars (range 4.8–7.5B). We also showed that these community-based HIV programs were cost-saving: from 2005 to 2011, every dollar invested in these programs saved about $5. This study is an important first step in understanding the impact of investing in community-based HIV prevention programs in Ontario and recognizing the impact that these programs have had in reducing HIV infections and health care costs.

## Resumen

En Ontario, inversiones de programas para prevención de VIH con base comunitaria, se han llevado acabo, durante las últimas dos décadas. Estos esfuerzos se han presumido como efectivos, aunque sin por el momento, se hayan realizado evaluaciones sistemáticas. Para ayudar a cerrar la ausencia de conocimiento, se condujo una evaluación a nivel macro, sobre la inversión de programas de prevención de VIH en Ontario, desde la perspectiva de los contribuidores de impuestos. Los resultados muestran que: desde 1987 hasta el 2011, los programas comunitarios de prevención provinciales ayudaron a prevenir un total de 16, 672 (nuevas) infecciones de VIH, ahorrándole al sistema de salud de Ontario aproximadamente $6.5 billones de dólares (rango: $4.8 B-$7.5 B). También mostramos que los programas comunitarios de prevención, desde el 2005 al 2011, ahorraron de cada dólar invertido, $5 dólares (CAD). Este estudio es un paso importante para el entendimiento del impacto de la inversión en programas con base comunitaria, y reconoce el impacto que estos programas han tenido en la reducción de infecciones de VIH y el costo en el cuidado de la salud, en Ontario.

## Introduction

The economic burden of HIV infection is substantial. In the United States, on average, the life-time cost per case is estimated as ranging from $253,000 to $402,000 (USD) [1]. Over the past two and a half decades, community-based programs and behavioural prevention strategies have played key roles in HIV and sexually transmitted infection prevention in Canada, the United States, Australia, the United Kingdom and elsewhere. A significant body of evidence has demonstrated that community-based and behavioural interventions are effective in: reducing risky sexual behaviour and the incidence of sexually transmitted infections in high-risk populations [2–7]; increasing condom use [8–10]; increasing knowledge of HIV transmission and prevention [7]; improving adherence to antiretroviral therapy [11]; and improving retention to care and treatment [6, 12]. Since the beginning of the HIV epidemic, Ontario’s HIV prevention strategies have relied heavily on province-wide, community-based HIV organizations to deliver prevention programs, educate populations and communities at-risk, and develop meaningful relationships with the community [13, 14]. In its prevention strategies, the province has strategically taken into account the impact of stigma, culture and human rights, as well as the social, behavioural and structural factors that affect HIV risk [13, 14].

Though many community-based behavioural HIV prevention programs have been shown to be successful, their outcomes often rely on self-reported behaviour change. The link between investments in these programs and the actual reduction of HIV infections is not well understood. However, understanding the link is vital for health planners and policy decision makers trying to determine whether allocating scarce resources to these programs is good value for money.

The major difficulty in understanding the impact of investment is the long causal pathway that runs between investments made and actual reductions in HIV infections. The impact of investment may take many years and, along the way, there are many confounding factors that might influence final outcome(s). Despite these difficulties, researchers at the U.S. Centers for Disease Control and Prevention (CDC) have created methodologies and models to evaluate the economic impact of HIV prevention programs and to provide support in recent years for funding resource allocation in the U.S. [15–21]. A correlation analysis showed that U.S. investments in HIV prevention appear to be correlated with the reduction of HIV incidence from 1985 to 2006 [22]. Another U.S. analysis found that investments in HIV prevention programs between 1991 and 2006 helped to avert 361,878 new HIV cases and saved the U.S. health care system $130 billion [17]. A model-based evaluation study has shown that most non-biomedical, community-based and behavioural interventions are cost saving [23]. An optimal portfolio of cost-saving interventions could reduce new HIV cases by 34 % and save $5 billion (USD) over the next 20 years [23]. Substantially scaling up cost-effective HIV prevention programs in the future may help avert new HIV cases and reduce costs in the U.S. and other countries [18–21].

To our knowledge, no similar evaluation analysis has been done for province-wide, community-based programs for HIV prevention in Ontario; there has also been no analysis of the level of investment required to have an impact. We took a first step to evaluate community-based HIV prevention programs and strategies at the macro level in the social and political context of Ontario, Canada. We did this by: retrospectively quantifying the historical impacts of community-based programs for HIV prevention in Ontario from 1987 to 2011; estimating the economic impacts of these programs from the payer perspective; and evaluating the financial return on investment of Ontario’s community-based programs for HIV prevention.

## Materials and Methods

To evaluate the historical and economic impacts of Ontario’s community-based programs, we applied well-established methods used to evaluate the publicly-funded U.S. investments in HIV prevention strategies [15–21]. This study was based on aggregate data, so did not require institutional ethical approval. All data used in this study were obtained through approvals from the provincial and federal governments.

### Ontario’s Province-Wide Community-Based HIV Programs

In Ontario, community-based HIV organizations are non-profit organizations governed by independent boards, established specifically to provide HIV prevention, education, outreach and support services to people living with HIV or to populations at risk of HIV (e.g., gay men, people who inject drugs, people from countries where HIV is endemic, and Indigenous people). It is important to include funding for support services for people living with HIV within prevention programming because of the critical impact that education and support have on onward transmission. Table 1 describes the major key non-biomedical and behavioural interventions for HIV and sexually transmitted infections (STIs) provided by community-based HIV organizations in Ontario. It also cites the literature on the effectiveness of each type of intervention. It is worth noting that the services offered by Ontario’s community-based HIV organizations differ from the HIV/STI prevention services offered by the province’s public health units, which focus mainly on providing sexual health clinics for the general population, testing services, and tracing the contacts of people newly diagnosed with HIV or another STI. While both community-based HIV organizations and public health units distribute condoms, public health condom distribution programs are focused mainly on youth with a primary goal of preventing STIs and teenage pregnancy, while community-based HIV organizations focus on condom distribution to populations most at risk of HIV.

**Table 1:** Main non-biomedical interventions for HIV and sexually transmitted infection prevention provided by community-based AIDS service organizations and a summary of corresponding evidence of the effectiveness of these interventions

Non-biomedical intervention	Target populations	Examples of Ontario’s community-based programs	Evidence of effectiveness in current literature
Individual-, group-, and community-level education, outreach, and community campaigns that promote risk-behaviour reduction	People living with HIV Gay, bisexual and other men who have sex with men Indigenous people African, Caribbean and Black Ontarians Women at-risk (who engage in activities with a high risk of exposure to HIV, or with priority populations)	Prevention/education activities targeted to high-risk populations (e.g., group workshops/presentations in varying locations and one-on-one education through outreach activities) Community campaigns such as the HIV Stigma campaign, Our Agenda, Keep It Alive for African, Caribbean and Black communities etc.	Reduce odds of unprotected anal intercourse by 27–43 %, and increase odds of condom use by 81 % [8–9]
Promote HIV medication adherence	People living with HIV	Clinical counseling Case management Support sessions for HIV symptoms management, treatment/medication Adherence	Increase HIV medication adherence behaviour and reduce HIV viral loads among people living with HIV [11]
Social support and social inclusion	People living with HIV People affected by HIV (e.g. family, friends, partners) People at risk for HIV	Clinical counseling Support sessions for disclosure, emotional well-being, harm reduction, physical health, employment services, relationships/social supports, financial counseling, housing, stigma/discrimination, employment services, wellness checks Bereavement services	A high level of social support is associated with fewer risky sexual behaviours [5] Support programs can achieve a high level of retention in care (91.4 %) over a 4-year follow-up [6]
Needle and syringe programs, safer inhalation programs, harm reduction outreach programs, and community development for people who use drugs	Injection drug users / people who use drugs	Distribution of safer injection equipment: cookers, filters, needles, sharps containers, swabs, ties/tourniquets, vitamin C/acidifiers, water for injection, and safer inhalation equipment Harm reduction activities, outreach and education programs (e.g. safer injection, safer inhalation, and safer sex practices to reduce HIV risk and transmission)	Exposure to needle and syringe programs was associated with reduction of HIV transmission (pool effect size: 0.4, 95 % CI 0.22–0.81) [2] Community outreach programs for injection drug users were effective [4]
Education and support for reduction of risky HIV transmission behaviour	People living with HIV Gay, bisexual and other men who have sex with men Indigenous people African, Caribbean and Black Ontarians People who use drugs Women at-risk (who engage in activities with a high risk of exposure to HIV, or with priority populations)	Prevention, education, and outreach activities	Education and support programs significantly reduced unprotected sex (OR 0.57, 95 % CI 0.40–0.73), and incidence of sexually transmitted disease (OR 0.20, 95 % CI 0.05–0.73) [3] Men living in geographic regions of Ontario with HIV prevention programming had unprotected homosexual intercourse with both casual and regular partners significantly less frequently [60]
Mass media programs for HIV prevention	People living with HIV Gay, bisexual and other men who have sex with men Indigenous people African, Caribbean and Black Ontarians People who use drugs Women at-risk (who engage in activities with a high risk of exposure to HIV, or with priority populations)	Mass media campaigns, e.g. Keep It Alive, HIV Stigma, Our Agenda	Increases in condom use [effect size (d):0.25, 95 % CI 0.18–0.31], knowledge of HIV transmission (d: 0.30, 95 % CI 0.18–0.41), knowledge of HIV prevention (d: 0.39, 95 % CI 0.25–0.52) [7]
Mass condom, and safer sex materials distribution	People living with HIV Gay, bisexual and other men who have sex with men Indigenous people African, Caribbean and Black Ontarians People who use drugs Women at-risk (who engage in activities with a high risk of exposure to HIV, or with priority populations)	Distribution of condoms, lubricant and dental dams	Increased condom use (OR 1.8, 95 % CI 1.51–2.17), condom acquisition (OR 5.4, 95 % CI 1.86–15.66), and reduced incidence of sexually transmitted disease (OR 0.69, 95 % CI 0.53–0.91) [10]
Practical assistance programs (includes distribution of practical assistance items)	People living with HIV Gay, bisexual and other men who have sex with men Indigenous people African, Caribbean and Black Ontarians People who use drugs Women at-risk (who engage in activities with a high risk of exposure to HIV, or with priority populations) People affected by HIV	Practical assistance programs, e.g. access to food programs, access to complementary therapies, emergency financial assistance, assistance accessing provincial drug payment programs, child care subsidy, clothing, household items, help with transportation, assistance with tax, insurance, or legal information	Support program can achieve a high retention of care (91.4 %) over a 4-year follow-up [6]
Linkage to HIV care, Ontario’s testing programs, HIV supportive case management, and clinical counseling	People living with HIV	Case management Support sessions that focus on connection to HIV care, retention in HIV care, HIV management, supporting clients to make/keep/or travel to medical appointments, referrals to HIV care, testing or treatment, or accompanying clients to medical appointments. Anonymous point-of-care testing as well as standard blood draw HIV testing—anonymous, nominal, non-nominal/coded Clinical counseling services	Case management and community engagement program increased likelihood of retaining in care (OR 4.13, 95 % CI 1.93–8.85) [12] Community-based approach increased uptake of HIV testing and counseling (RR 10.65, 95 % CI 6.27–18.08) and increased proportion of first-time testers (RR 1.23, 95 % CI 1.06–1.42) [55] Support programs can achieve a high retention of care (91.4 %) over a 4-year follow-up [6]
Increase awareness of use of post-exposure prophylaxis (PEP)	People living with HIV Gay/bi/trans and other men who have sex with men	Education activities include group workshops/presentations and one-on-one education through outreach targeted to priority populations Support sessions provided to clients at Ontario’s community-based HIV organizations	Increased use of PEP for HIV by 42 % [62]
Supportive housing	People living with HIV	HIV supportive housing programs across Ontario: Fife House & McEwan Housing and Support Services (Toronto), AIDS Niagara (St. Catherines), Bruce House (Ottawa), John Gordon Home-Regional HIV/AIDS Connection (London), and Abercrombie Place-ARCH (Guelph)	Homeless/ marginally-housed people living with HIV were associated with poorer HAART access/adherence or treatment outcomes [63] Housing status reduces needle sharing (OR 0.37, 95 % CI 0.15–0.81) and unprotected sex (OR 0.39, 95 % CI 0.18–0.84) [64]

In Ontario, community-based HIV organizations are primarily funded by the AIDS Bureau of the Ontario Ministry of Health and Long-term Care (since 1987) and the AIDS Community Action Program (ACAP) of the Public Health Agency of Canada (since 1993). Community-based HIV organizations also seek funding from other federal, provincial, regional and local government health programs, from foundations, and from the general public [13, 14]. In general, about 58 % of their funding comes from the AIDS Bureau, 8 % from ACAP, 21 % from other government health programs and 13 % from other sources [13, 14].

### Data Sources and Analytic Framework

Our analytic framework was based on well-established methods used to evaluate U.S. investments in HIV prevention strategies [15–21]. Analyses were conducted from the payer perspective. Ontario HIV transmission rates were estimated from Ontario HIV surveillance reports dating to 1986, the first full year in which HIV testing results became available [24]. Lifetime HIV treatment costs in Canada were obtained from the scientific literature [25]. Expenditures of community-based HIV prevention programs were obtained from budgetary costs submitted by all community-based organizations [13]. All estimated costs are reported in 2011 Canadian dollars using the corresponding consumer price indices (CPI) [26] and a discounted rate of 3 % [27, 28].

We first quantified the historical impacts of community-based HIV programs by estimating the number of HIV cases averted and the associated estimated cost savings to the Ontario health care system. We then estimated the financial return-on-investment ratio for Ontario’s investment in community-based HIV programs from 2005 to 2011.

In our analysis, we recognize that community-based HIV prevention programs are not the only interventions that influence infection rates. General programs offered by public health units contribute as do biomedical treatment programs (i.e., the use of antiretroviral therapy, which reduces infectiousness).

To estimate the *number of HIV infection cases averted by province*-*wide community*-*based HIV programs*, we first used the differential in HIV transmission rates between Ontario and a comparator (which was assumed to not have Ontario’s HIV prevention programs) to estimate the total number of HIV infections averted by both biomedical (i.e., antiretroviral therapy-related) and non-biomedical (i.e. community-based and public health) interventions in Ontario. The HIV transmission rate of the comparator was assumed to be the mean of Ontario’s incidence rates from 1987 (when community-based and public health programs began) to 1996 (1 year before highly active antiretroviral therapy [HAART] became available). The comparator was Ontario itself—only assuming a period with no HIV prevention programs or very minimal programming—so that systematic differences between Ontario and the comparator were minimized. Our assumption of the comparator’s HIV transmission rate was conservative because we used average rates from a period when public health and community-based programs were already in place.

Once we had the total number of HIV cases averted by Ontario’s HIV prevention programs, we factored in the number of cases averted by type of intervention based on literature-based proportions during both the pre-HAART and post-HAART eras. During the pre-HAART era (before 1997), we assumed that all the HIV infection cases averted were due to province-wide community-based and public health programs. We also assumed (conservatively) that 30 % of the HIV cases averted were due to the effort of Ontario’s STI public health control programs, although a recent mathematical model has found that the probability of HIV infections averted due to STI preventions (cofactors and STI screening) was about 15 % [23, 29].

During the post-HAART era (1997 and onwards), we assumed that biomedical interventions were responsible for averting 75 % of new infections while non-biomedical programs (public health and community-based programs) helped to avert 25 % of new diagnoses. Our assumptions are very conservative because the effect of the introduction of antiretroviral therapy on HIV prevention in developed countries was estimated at 25 % [23, 30] and the theoretical efficacy (universal annual screening, immediate linkage to care, universal ART and perfect adherence) of implementing HAART-related interventions is unlikely to be achieved in real world settings. In addition, a recent mathematical model demonstrated a few counter-factual scenarios in a simple world for HIV epidemics based on having only ART and condoms (the key representation of non-ART intervention) for HIV prevention. If no ART were introduced and all condom use ceased, the mean HIV incidence would have increased by 0.36 and 2.25 respectively [30]. These scenarios give a rough idea that approximately 14 and 86 % of the total decrease of HIV incidence was attributed to ART introduction and to condom use respectively [30]. For the total number of cases averted by non-biomedical interventions, we assumed that about 30 % were averted by the efforts of public health programs for STI prevention.

We estimated *savings in Canadian health care system costs* by multiplying the number of HIV infection cases averted (attributed to community-based HIV programs) by the lifetime treatment cost associated with HIV infection. Annual direct costs for treating HIV were estimated by conducting a literature review for cost-of-illness studies published between January 1, 1986 and December 31, 2012 using HealthSTAR, Medline, PubMed, EMBASE, Cochrane databases and grey literature (i.e., government or non-government reports, theses, dissertations, and conference proceedings). Search terms included: “cost of illness,” “costs,” “health expenditures,” “cost analysis,” “economic value of life,” “direct cost,” and “medical cost.” We included studies: (a) with HAART as a routine clinical treatment; (b) from the Canadian health care system; and (c) concerning people living with HIV in general. Seven academic articles [31–35] and grey literature reports [36, 37] were found. Four articles [31, 32, 34, 35] were not related to estimating direct medical costs and the methods in two reports [36, 37] were not rigorous. Our estimate for the direct costs to treat HIV was based on a retrospective cohort study conducted in Alberta, Canada by Krentz et al. [33]. The mean treatment cost per patient per month (PPM) was estimated as $1,159 (in 2005 Canadian dollars), varying from $979 to $2,687 PPM depending on the stage of HIV infection [33]. Therefore, using the corresponding CPIs, the mean treatment cost per patient per year was estimated as $13,908 (in 2005 Canadian dollars) (calculated as $1,159 × 12 months). Considering the type of drug therapy and CD4 counts when first infected, the number of years on ART was assumed to be between 19 and 32 years from the time of infection [1, 38, 39]. Using a discount rate of 3 % [27, 28], the present value of the mean lifetime treatment cost for HIV infection in Canada was approximately $256,090 per patient in 2005 Canadian dollars, varying from $213,123 to $297,475. Using the corresponding CPIs [26], the present value of the mean lifetime treatment cost for HIV infection is $286,965 in 2011 Canadian dollars, varying from $238,817 to $333,339.

We estimated the *financial return*-*on*-*investment (ROI) ratio* [40, 41] *for Ontario’s investments in community*-*based HIV programs* (from 2005 to 2011) by dividing the net present value of net savings in health care costs from averted HIV cases by the total investments in community-based HIV programs, in 2011 dollars, discounted at 3 %. Completed budgetary data for community-based HIV programs were not available prior to 2005.

### Sensitivity Analyses

Several one-way sensitivity analyses were conducted to examine the impact of assumptions made in analyzing the number of HIV infections averted and the savings attributed to community-based HIV programs in the health care system. First, we assessed the impact by assuming that the HIV transmission rate of the comparator was the largest or the smallest transmission rate from 1987 to 1996 (the pre-HAART era) in Ontario. Second, we reduced our assumed proportion of averted HIV infections that were attributed to public health STI programs from 30 % to a more realistic proportion of 15 % [23, 29]. Third, we reduced our assumed proportion of the averted HIV infections that were attributed to the introduction of antiretroviral therapy from 75 % to a more realistic proportion of 25 % [23, 30]. Fourth, we assessed the impact by assuming the discount rate of 5 % for deriving the present value of lifetime HIV treatment costs. Finally, we simultaneously varied two or more of our assumptions to examine the impact on our results.

## Results

**Fig. 1:**
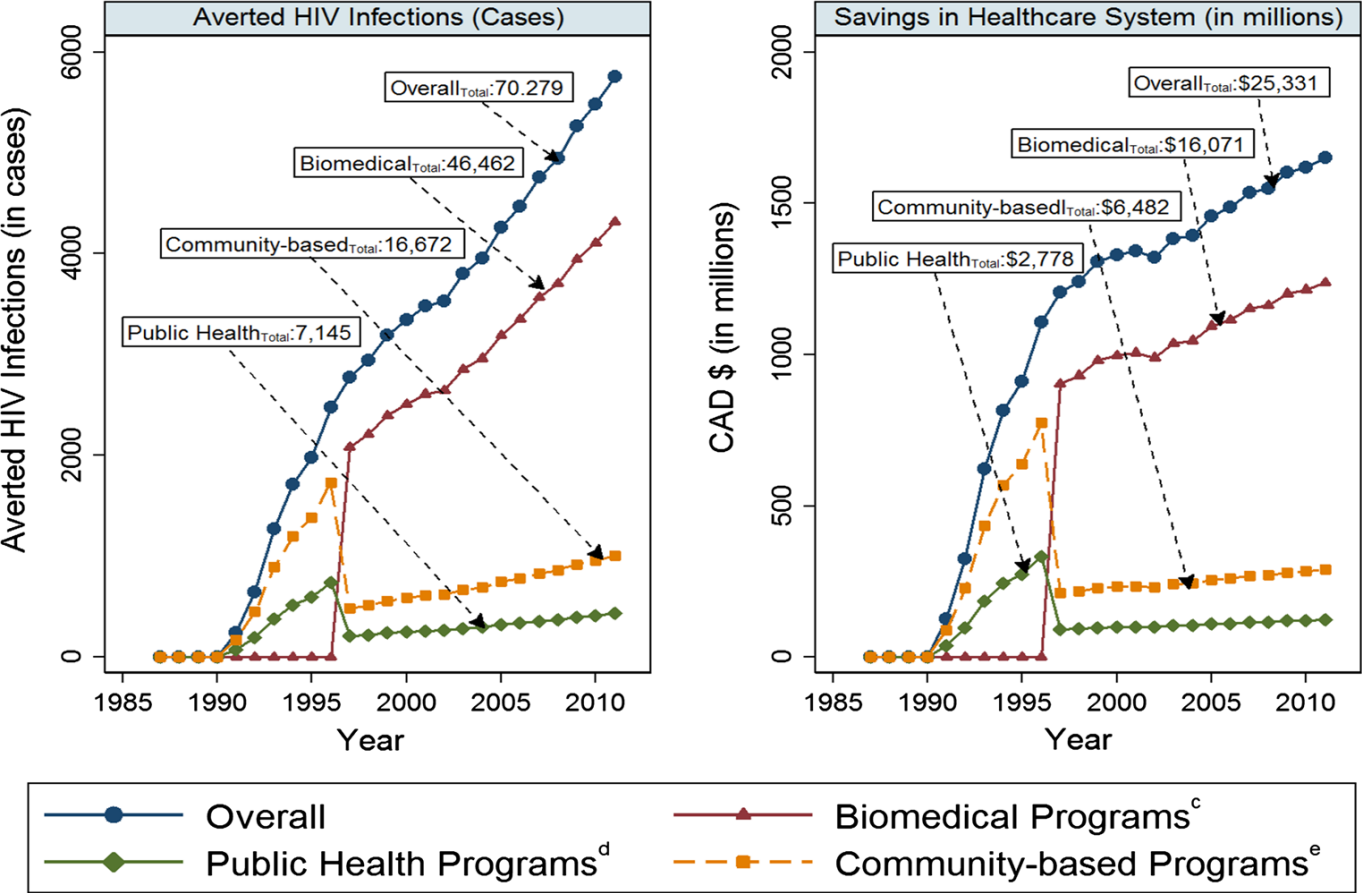
Estimated number of HIV infections averted^a^ and savings to the Canadian health care system (in millions)^b^ by intervention type in Ontario (1987–2011**).** *Notes* (a) number of HIV infection cases averted by province-wide community-based HIV prevention programs was estimated in two steps. We first used the differential in HIV transmission rates between Ontario and a comparator to estimate the total number of HIV infections averted by both biomedical (i.e. antiretroviral therapy-related) and non-biomedical (i.e. community-based and public health program) interventions in Ontario. The HIV transmission rate of the comparator was assumed to be the mean of Ontario’s incidence rates from 1987 to 1996 (when investments in community-based and public health programs had begun but HAART was not yet in place). Once we had the total number of HIV cases averted by the Ontario’s HIV prevention programs, we factored in the number of cases averted by type of intervention based on literature-based proportions. We assumed conservatively that 30 % of the HIV cases averted were due to the effort of Ontario’s STI public health control programs (although results of a recent mathematical model suggest that the probability of HIV infections averted due to STI preventions [cofactors and STI screening] was about 15 %) [23, 29]. During the post-HAART era (1997 and onwards), we assumed the proportion of HIV infection cases averted by biomedical programs as 75 % of the total number of the cases averted and by non-biomedical programs (public health and community-based programs) as 25 % of the total cases averted [23, 30]. Our comparator was Ontario itself, so systematic differences between Ontario and the comparator were minimized. Our assumption of the comparator’s HIV transmission rate was conservative because we used average rates during a period when public health and community-based programs were already in place. (b) Savings in Canadian healthcare system costs were estimated by multiplying the number of HIV infection cases averted (attributed to community-based HIV programs) by the lifetime treatment cost associated with HIV infection. Mean lifetime medical costs were estimated as $286,965 (range $238,817–$333,339) per patient in 2011 Canadian dollars. All estimated savings are reported in 2011 CAD using the corresponding consumer price indices in Ontario [26]. (c) Biomedical interventions were assumed to be related to the introduction of antiretroviral therapy. (d) Public health programs were assumed to be mainly focused on sexually transmitted infections (STIs). These programs included a wide range of initiatives, e.g. medications to treat STIs, education and outreach programs, needle exchange programs, sexual health hotlines, etc. (e) Community-based programs were assumed to be non-biomedical interventions excluding public health sexually transmitted infection control programs [26]

Table 2 presents the estimated number of new HIV cases averted by all types of HIV prevention strategies in Ontario from 1987 to 2011. Overall, during this period and in contrast to the comparator (without HIV prevention strategies in place), we estimated that approximately 70,279 new HIV infection cases were averted by Ontario’s HIV prevention strategies. When stratified by intervention type, we estimated that about 46,462, 7145 and 16,672 new HIV cases were averted by biomedical, public health, and community-based HIV programs respectively during this period (Fig. 1).

**Table 2:** HIV incidence, prevalence, and transmission rate and total number of HIV infections averted (in cases) in Ontario (1987–2011)

Year	Ontario			Comparator^a^	New HIV infection cases		Total HIV infection averted (C) = (A) − (B)
	HIV incidence (1)	HIV prevalence in previous year (2)	HIV transmission rate^b^ (3)	HIV transmission rate^b^ (4)	Comparator (A) = [(4) × (2)] ÷ 100	Ontario (B) = [(3) × (2)] ÷ 100	
1987	1546	1546	53.1	20.8	321.6	821.3	0.0
1988	1442	4352	33.1	20.8	905.3	1442.0	0.0
1989	1702	6054	28.1	20.8	1259.4	1702.0	0.0
1990	2062	8116	25.4	20.8	1688.3	2062.0	0.0
1991	1822	9938	18.3	20.8	2067.3	1822.0	245.3
1992	1797	11735	15.3	20.8	2441.1	1797.0	644.1
1993	1477	13212	11.2	20.8	2748.4	1477.0	1271.4
1994	1304	14516	9.0	20.8	3019.7	1304.0	1715.7
1995	1314	15830	8.3	20.8	3293.0	1314.0	1979.0
1996	1034	16864	6.1	20.8	3508.1	1034.0	2474.1
1997	924	17788	5.2	20.8	3700.3	924.0	2776.3
1998	953	18741	5.1	20.8	3898.6	953.0	2945.6
1999	889	19630	4.5	20.8	4083.5	889.0	3194.5
2000	886	20,516	4.5	20.8	4267.8	923.2	3344.6
2001	957	21,473	4.6	20.8	4466.9	987.8	3479.1
2002	1,132	22,605	5.2	20.8	4702.4	1175.5	3526.9
2003	1,164	23,769	4.8	20.8	4944.5	1140.9	3803.6
2004	1,085	24,854	4.9	20.8	5170.2	1217.8	3952.4
2005	1,106	25,960	4.4	20.8	5400.3	1142.2	4258.0
2006	1,132	27,092	4.3	20.8	5635.8	1165.0	4470.8
2007	1,049	28,141	3.9	20.8	5854.0	1097.5	4756.5
2008	1,102	29,243	3.9	20.8	6083.2	1140.5	4942.7
2009	999	30,242	3.4	20.8	6291.0	1028.2	5262.8
2010	1,023	31,265	3.3	20.8	6503.8	1023.0	5480.8
2011	946	32,211	2.9	20.8	6700.6	946.0	5754.6
				Total	99,055.1	30,628.9	70,278.9

^a^ HIV transmission rate of the comparator was assumed to be the mean of Ontario’s incidence rates from 1987 to 1996 [i.e., a time when the investments of community-based and public health programs had begun (1987) and before the introduction of highly active antiretroviral therapy (HAART) (1996)]. Our comparator was Ontario itself, so systematic differences between Ontario and the comparator were minimized. Our assumption of the comparator’s HIV transmission rate was conservative because we used average rates during a period when public health and community-based programs were already in place

^b^ Per 100 persons living with HIV

In terms of the corresponding economic impacts, we estimated that a total of $25.3 billion CAD (range $18.8–29.4 billion) (in 2011 dollars, 1 USD = 0.99 CAD [42]) was saved in the Ontario health care system (see Fig. 1) from 1987 to 2011. Based on type of intervention, about $16.1 billion CAD (range $11.9–18.7 billion), $2.8 billion CAD (range $2.1–3.2 billion), and $6.5 billion CAD (range $4.8–7.5 billion) were saved by biomedical, public health, and community-based HIV strategies respectively (see Fig. 1).

Table 3 presents the results of the financial ROI ratio for community-based HIV programs in Ontario from 2005 to 2011. Completed budgetary data for community-based HIV programs were not available prior to 2005. During the period from 2005 to 2011, approximately $328 million (in 2011 dollars, discounted by 3 %) were invested in community-based HIV programs and these programs helped to save about $1.9 billion (in 2011 dollars, discounted by 3 %). Thus, the financial ROI ratio was about 4.8 (i.e., a dollar invested in community-based HIV programs would save the Ontario health care system approximately $5 dollars in treatment costs).

**Table 3:** Financial return on investment from Ontario’s investment in community-based programs for HIV infections (2005–2011)

Year	Savings in Canadian health care system by community-based programs^a^	Investments in community-based HIV programs			
		AIDS Bureau^b^	ACAP^c^	Other governmental^d^	Others^e^
2005	$ 213,833,757	$ 12,644,074	$ 2,534,540	$ 8,118,491	$ 10,222,525
2006	$ 224,518,625	$ 13,607,252	$ 3,323,877	$ 9,532,182	$ 10,037,582
2007	$ 238,864,805	$ 13,830,988	$ 2,441,225	$ 9,948,038	$ 9,904,051
2008	$ 248,218,737	$ 16,567,895	$ 3,252,240	$ 11,584,297	$ 10,968,874
2009	$ 264,291,987	$ 17,698,104	$ 3,796,024	$ 12,058,316	$ 7,962,258
2010	$ 275,241,483	$ 20,083,701	$ 3,564,936	$ 14,223,361	$ 10,125,239
2011	$ 288,990,894	$ 20,338,042	$ 3,859,085	$ 13,106,427	$ 9,719,275
Total	$ 1,753,960,288	$ 114,770,056	$ 22,771,927	$ 78,571,112	$ 68,939,804
Present value of total savings in Canadian health care system (2005–2011) (in 2011 dollars, discounted by 3 %)^f^ (A)					$ 1,908,564,059
Present value of investments in community-based HIV programs (2005–2011) (in 2011 dollars, discounted by 3 %)^f^ (B)					$ 327,793,439
Financial return on investment ratio (C) = [(A) − (B)]/(B)					4.8

^a^ Savings in Canadian health care system costs were estimated by multiplying the number of HIV infection cases averted (attributed to community-based HIV programs) by the lifetime treatment cost associated with HIV infection. Mean lifetime medical costs were estimated as $286,965 (range $238,817–$333,339) per patient in 2011 Canadian dollars. All estimated savings are reported in 2011 CAD using the corresponding consumer price indices in Ontario [26]

^b^ AIDS Bureau of the Ontario Ministry of Health and Long-term Care (MOHLTC)

^c^ AIDS Community Action Program (ACAP) managed by the Public Health Agency of Canada Regional Offices

^d^ Other governmental funding includes other federal, provincial, regional, and municipal-level funding sources

^e^ Other sources includes charitable donations, fundraising, United Way, and others

^f^ Reported present value in 2011 dollars using the corresponding consumer price indices [26] and a discount rate of 3 %

**Fig. 2:**
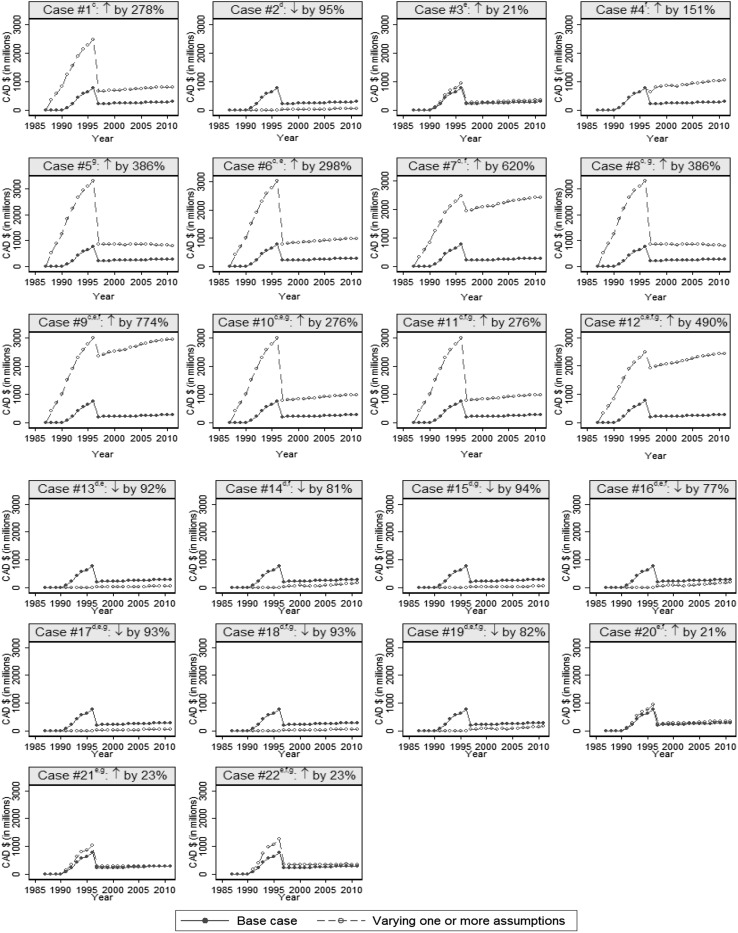
Sensitivity analyses^a^ for examining impacts on savings^b^ in Canadian health care systems attributed to community-based programs^c^ for HIV Prevention through varying one or more assumptions. *Notes* (a) Varies one or more assumptions simultaneously. (b) Medical system savings are calculated based on the estimated mean of lifetime medical costs, or $286,965 (range $238,817–$333,339) per patient in 2011 Canadian dollars. All estimated savings are reported in 2011 CAD using the corresponding consumer price indices in Ontario [26]. (c) Community-based programs were assumed to be non-biomedical interventions excluding public health sexually transmitted infection control programs. Non-biomedical interventions were assumed to be mainly education, outreach, and social behavioural programs that were not related to the introduction of antiretroviral therapy (e.g. condom distribution programs, community outreach, needle exchange programs, sexual education, programs for improving antiretroviral adherence or increasing awareness of the use of post-exposure prophylaxis, etc.). HIV transmission rate of the comparator was the largest transmission (53.1 per 100 persons living with HIV) rate from 1987 to 1996 (pre-HAART era) in Ontario. (d) HIV transmission rate of the comparator was the smallest transmission (6.1 per 100 persons living with HIV) rate from 1987 to 1996 (pre-HAART era) in Ontario. (e) A more realistic proportion (15 %) of averted HIV infections were assumed to be attributable to public health STI programs [23, 29]. (f) We assumed the effect of the introduction of antiretroviral therapy on HIV prevention at a realistic proportion of 25 % [23, 30]. (g) We assumed the discount rate of 5 % for deriving the present value of lifetime HIV treatment costs

Figure 2 presents the results of a series of sensitivity analyses for the impacts on health care savings attributed to community-based HIV programs. The results were sensitive to our assumptions. By assuming that the HIV transmission rate of the comparator was the largest HIV transmission rate in Ontario during the pre-HAART era, the health care savings increased by approximately 278 % (see case #1). Second, assuming that the HIV transmission rate of the comparator was the smallest HIV transmission rate in Ontario during the pre-HAART era, the savings were reduced by approximately 95 % (see case #2). Third, by assuming a more realistic proportion (15 %) of averted HIV infections attributed to public health STI programs, the savings increased by 21 % (see case # 3). Fourth, by assuming the effect of the introduction of the antiretroviral therapy on HIV prevention at a more realistic proportion of 25 %, the savings were increased by approximately 151 % (see case #4). Fifth, when assuming the discount rate of 5 % for deriving the present value of the lifetime treatment cost for HIV, the savings increased by 386 % (see case #5). Finally, by changing two to three assumptions simultaneously, the changes in savings attributed to community-based HIV programs varied from −95 % to +774 % (see cases #6–22).

## Discussion

Our macro-level analysis is the first study to attempt to quantify the historical and economic impacts of province-wide, community-based programs for HIV prevention in Ontario. From 1987 to 2011, our results show that these programs might have helped to avert approximately 16,672 new cases of HIV infection, and saved a total of $6.5 billion (range 4.8–7.5B) for the Ontario health care system (in 2011 Canadian dollars). We also showed that community-based HIV programs were cost-saving because every $1 invested in these programs from 2005 to 2011 saved about $5 in treatment costs. Nonetheless our evaluation was conducted at macro-level. It did not attempt to assess which types of community-based prevention interventions were most effective.

Our results are consistent with similar studies in other jurisdictions. Our projection of the number of HIV infections that would occur if HIV prevention interventions were not in place is comparable to the U.S. CDC estimates [17–21]. In Ontario, the HIV prevention strategy has relied heavily on community actions and programs since the beginning of the epidemic [43]. Similarly, Australian researchers have found that community-based HIV prevention programs/actions have had a remarkable impact on reducing HIV incidence [44]. Community-based HIV prevention in Australia has been credited as a “public health milestone of global importance” in reducing HIV cases in the past two decades [44]. This type of data on the cost saving potential of investments in community-based programs may be particularly helpful to the Ontario health care system, which—unlike the mixed private–public funded system in the U.S.—is predominantly a single (government) payer system. Savings from investments in prevention programs accrue back to system, where they can be reallocated to other health priorities.

Our results for the financial ROI ratio for community-based HIV programs were comparable to findings of ROI ratios in mental health promotion programs and other public health strategies, which ranged from $1 to $25 [45–47]. Our ROI estimate was conservative because we only included savings in direct health care costs as opposed to the broader health and social benefits of averted HIV infections.

Over the past 28 years, Ontario’s community-based HIV programs have made significant efforts to reach populations and communities at risk, and to develop the kind of meaningful relationships and programs that lead to good health outcomes, including a reduction in new infections [13, 14]. Even in the post-antiretroviral therapy era, with its strong focus on treatment-related prevention, more and more evidence is showing that biomedical and social behavioral interventions are essential complements to each other in order to deliver effective combination prevention programs [9, 23, 30, 48–51]. Social behavioural and structural interventions, together with community actions, are important at each step to help people living with HIV achieve virologic suppression, including interventions that respond to stigma, engage patients in HIV testing, initiate ARV medication, maintain long-term adherence to ARV, and retain patients in HIV care [49–53]. Given the comprehensive nature of community-based HIV prevention programs in Ontario and the emerging evidence on the effectiveness of combination approaches to prevention, our findings are likely a conservative estimate of the impact of Ontario’s investment in community-based programs for HIV prevention.

Our study has several limitations. First, the economic evaluation analyses relied on aggregate-level data and the ecological analysis might not have allowed our analysis to consider heterogeneity among individuals or to control for other confounding factors over time. However, these types of methods and analytic approaches have been previously used in many U.S. studies to successfully evaluate their HIV prevention programs and strategies [15–21].

Second, we made the causal assumption in our analysis that community-based HIV prevention programs in Ontario can reduce new HIV infections. However, this is a reasonable assumption given that many of the community-based HIV prevention programs in Ontario are evidence-based, and the effectiveness of these types of programs have been shown in the literature as summarized in Table 1 [2, 4, 5, 7, 8, 54–60]. In addition, an outcomes evaluation report, based on sound qualitative methods, found that Ontario’s community-based HIV programs are effective in reaching vulnerable and marginalized populations, increasing awareness and knowledge of HIV transmission and consequences, reducing risky behaviour for HIV infection, and increasing linkage to HIV care, support and treatment [14]. The report also concluded that successful HIV prevention outcomes are likely to be achieved by effective collaboration among healthcare providers, social services and community-based programs [14]. Future research should assess the effectiveness and cost-effectiveness of specific prevention programs at the individual level in community-based settings.

Third, we compared the yearly HIV transmission rate to the mean of Ontario’s incidence rates from 1987 to 1996 in order to estimate the total number of HIV infections averted by both biomedical (i.e. antiretroviral therapy-related) and non-biomedical (i.e. community-based and public health program) interventions in Ontario. Our comparator is a reasonable and conservative choice. During this period, investments in community-based and public health programs had begun and HAART was not yet in place. As well, our comparator was Ontario itself, so systematic differences between Ontario and the comparator were minimized.

Fourth, our results were shown to be sensitive to our assumptions. By changing one or more assumptions, the changes in saving attributed to community-based HIV programs varied from −95 to +774 % (see Fig. 2). Nevertheless, all negative changes in our savings estimate were due to the fact that we varied the HIV transmission rate of the comparator to the lowest HIV transmission rate in Ontario during the pre-HAART era (see Table 2). However, the lowest HIV transmission rate occurred in 1995 (see Table 2), when most of public health and community-based HIV programs were well-established. In addition, our assumption of the comparator’s HIV transmission rate was conservative because we used average rates from a period when public health and community-based HIV programs were already in place. Furthermore, as already discussed, we assumed the proportion of HIV infection cases averted by biomedical programs at the theoretical efficacy rate of 75 %, which is high given that the estimated effect of the introduction of antiretroviral therapy in the real world is 25 % [23, 30]. Moreover, we assumed that 30 % of the HIV cases averted were due to the effort of Ontario’s STI public health control programs, even though the results of a recent mathematical model suggest a rate of about 15 % [23, 29].

Despite these limitations, our results shed some light on investments made in community-based HIV prevention programs in Ontario and how these investments have reduced HIV infections and health care system costs. Given that an effective vaccine for HIV is not yet available [61] and that the HIV epidemic is far from over [24, 25], continued investments in a combination of effective and evidence-based programs is essential, particularly in the post-HAART era. A more holistic approach that strategically and effectively combines evidence-based community actions, public health, biomedical, structural, and socio-behavioural efforts will likely yield the most significant impact on reducing new HIV infections [49–53].

Future research should provide individual-level economic evidence of the impact of investments in community-based HIV prevention programs to help both provincial and federal governments make strategic decisions about how to have the most impact on the HIV epidemic in Ontario, especially in an environment of constrained health care resources.
